# Obstructive Azoospermia Revealing CFTR-Related Disorder in an Adult With Recurrent Pancreatitis

**DOI:** 10.7759/cureus.112411

**Published:** 2026-07-10

**Authors:** Hector R Gonzalez-Carranza, Gonzalo Montero-Sanchez, Lisandro C Vazquez-Niño, Luis A Reyes-Vallejo

**Affiliations:** 1 Department of Urology, Hospital Angeles Metropolitano, Mexico City, MEX; 2 Department of Urology, UMAE Hospital de Especialidades No. 25, IMSS, Monterrey, MEX; 3 Department of Urology, Hospital Regional Lic. Adolfo Lopez Mateos, ISSSTE, Mexico City, MEX

**Keywords:** cftr mutation, congenital bilateral absence of vas deferens, cystic fibrosis, gene mutations, male factor infertility

## Abstract

Obstructive azoospermia represents a critical diagnostic category in male infertility, with congenital bilateral absence of the vas deferens being a well-established etiology associated with Cystic Fibrosis Transmembrane Conductance Regulator (CFTR) gene variants. We report a 38-year-old man presenting with primary infertility and a history of recurrent pancreatitis. Evaluation revealed azoospermia with low ejaculate volume, normal hormonal profile, and bilateral absence of the vas deferens on physical examination. Genetic testing identified compound heterozygosity for a pathogenic CFTR variant (p.Phe508del) and a c.1210-34TG(12)T(5) splicing variant. The TG[n]T[m] polymorphic tract influences CFTR mRNA splicing, and the combination of T5 with longer TG repeats is associated with reduced CFTR function. This case underscores the importance of thorough urologic evaluation in patients with infertility and highlights the clinical relevance of less frequently emphasized variants such as TG12T5 in CFTR-related disorders.

## Introduction

Male infertility affects up to 15% of couples, with azoospermia accounting for approximately 1% of all men and up to 10-15% of infertile males. Differentiating obstructive from non-obstructive azoospermia is critical, as it significantly impacts both diagnostic evaluation and management. Congenital bilateral absence of the vas deferens (CBAVD) is a well-recognized cause of obstructive azoospermia and is strongly associated with mutations in the Cystic Fibrosis Transmembrane Conductance Regulator (CFTR) gene [1,2,3].

Although classically linked to cystic fibrosis (CF), CFTR-related disorders may present in adulthood with isolated or atypical manifestations, including infertility or recurrent pancreatitis. Symptomatic pancreatitis is uncommon in CF overall, affecting approximately 1-2% of patients, but it is more frequent in pancreatic-sufficient individuals, with reported rates of 10-20%. Among CF patients who develop pancreatitis, recurrent episodes or progression to chronic pancreatitis may occur in nearly 70% of cases. Recognizing these presentations is essential for urologists, as diagnosis has implications beyond fertility, including systemic disease evaluation and genetic counseling [4,5].

CFTR variants are detected in the majority of men with CBAVD, with reported rates of approximately 65-80% depending on ethnicity, mutation panel, and whether intronic variants such as the 5T/TG tract are included. In a meta-analysis, 78% of CBAVD patients carried at least one CFTR variant, while 46% had biallelic variants [6].

We present a case in which obstructive azoospermia led to the diagnosis of a CFTR-related disorder in an adult male with a history of recurrent pancreatitis.

## Case presentation

A 38-year-old man presented for evaluation of primary infertility after one year of unsuccessful attempts to conceive. The female partner had been evaluated by a gynecologist with no abnormalities identified. He had previously been evaluated by multiple urologists without a definitive diagnosis or improvement.

His medical history was notable for three prior episodes of acute pancreatitis since 2022, all treated conservatively without complications in different hospitals. He denied any known genetic disorders. Family history was significant for colorectal cancer in his maternal grandfather and gastric cancer in his paternal grandmother. He denied other relevant medical conditions.

The patient had no respiratory, sinus, gastrointestinal, or other symptoms suggestive of classic cystic fibrosis, and his nutritional status was not clinically altered. Physical examination revealed normal male secondary sexual characteristics. Testicular size and consistency were within normal limits, and external genitalia were unremarkable. However, the bilateral vas deferens were not palpable.

A semen analysis demonstrated azoospermia with low ejaculate volume, confirming the absence of spermatozoa despite centrifugation (Table 1). Hormonal evaluation, including follicle-stimulating hormone (FSH), luteinizing hormone (LH), testosterone, and prolactin, was within normal limits (Table 2), supporting a diagnosis of obstructive azoospermia.

**Table 1:** Semen analysis results. Azoospermia with low ejaculate volume, suggestive of obstructive etiology.

Parameter	Result	Reference range
Volume	0.2	≥1.5 mL
pH	6.5	≥7.2
Sperm concentration	0 sperm/mL	≥15 million/mL
Total sperm count	0	≥39 million
Fructose	Negative	Positive
Leukocytes	0	0-1 million/mL

**Table 2:** Hormonal profile. Normal hormonal profile consistent with obstructive azoospermia. FSH: follicle-stimulating hormone; LH: luteinizing hormone.

Hormone	Result	Reference range
FSH	3.6 mIU/mL	1.5-12.4 mIU/mL
LH	3.6 mIU/mL	1.7-8.6 mIU/mL
Testosterone	3.08 ng/mL	2.5-8.4 ng/mL
Prolactin	10.4 ng/mL	4.6-21.4 ng/mL
Estradiol	20.3 pg/mL	10-40 pg/mL

Given the absence of the vas deferens on physical examination, further evaluation with genetic testing was pursued. Analysis of the CFTR gene revealed two variants in heterozygosity: a pathogenic variant (p.Phe508del) and a TG12T5 variant associated with abnormal splicing; this combination is associated with CFTR-related disorders, including CBAVD. The TG12T5 allele results in reduced CFTR mRNA splicing efficiency, producing lower levels of functional CFTR protein. When co-inherited with a pathogenic variant such as p.Phe508del, the combined reduction in CFTR function reaches a threshold sufficient to manifest CBAVD and pancreatic dysfunction. (Table 3).

**Table 3:** CFTR genetic findings. Compound heterozygosity suspected associated with CFTR-related disorders including CBAVD. CBAVD: congenital bilateral absence of the vas deferens; CFTR: Cystic Fibrosis Transmembrane Conductance Regulator.

Variant	Type	Clinical significance
p.Phe508del	In-frame deletion	Pathogenic
TG12T5	Splicing variant	Disease-associated variant

**Figure 1:**
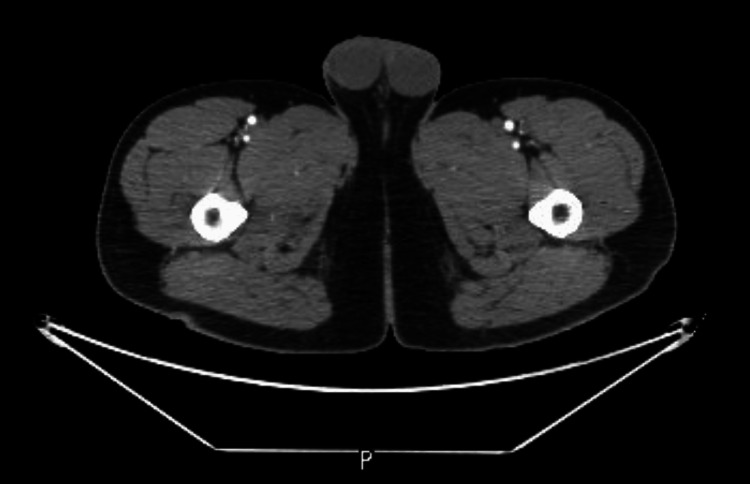
Pelvic computed tomography (CT) of the scrotum demonstrating normal testicular morphology. Pelvic CT scan imaging of the scrotum demonstrates bilaterally symmetric testes with preserved size and homogeneous parenchymal attenuation without evidence of focal lesions or structural abnormalities. This image was included to emphasize that normal-appearing testes do not exclude clinically relevant causes of azoospermia, including obstructive or congenital etiologies such as congenital bilateral absence of the vas deferens. Therefore, further diagnostic evaluation remains warranted despite preserved testicular morphology. CT: computed tomography.

Additional imaging studies performed for evaluation of pancreatic disease demonstrated calcifications, ductal dilatation, and atrophy, consistent with chronic pancreatic involvement, defined by the occurrence of two or more distinct episodes of acute pancreatitis separated by complete or near-complete clinical resolution. Scrotal imaging demonstrated normal testicular morphology without structural abnormalities (Figure 1).

Based on the clinical, laboratory, and genetic findings, a diagnosis of CFTR-related disorder consistent with a mild or atypical form of CF was established.

The patient received comprehensive counseling regarding the diagnosis of a CFTR-related disorder, including its implications for fertility, potential reproductive options, and the risk of transmitting CFTR variants to offspring. Although assisted reproductive techniques, including surgical sperm retrieval combined with intracytoplasmic sperm injection, were discussed, the patient elected not to pursue fertility treatment at that time.

## Discussion

This case illustrates a stepwise diagnostic pathway in which each clinical finding redirected the evaluation toward an underlying CFTR-related disorder. The initial finding of azoospermia with low ejaculate volume in the setting of normal testicular size and a normal hormonal profile made primary testicular failure less likely and supported an obstructive etiology. Careful physical examination then became decisive, as bilateral absence of the vas deferens shifted the differential diagnosis toward cCBAVD and prompted CFTR genetic testing. The patient’s history of recurrent pancreatitis, although not initially the reason for urologic referral, provided an important systemic clue that the infertility was not an isolated reproductive finding but part of a broader CFTR-related phenotype [7].

In men with azoospermia, distinguishing between obstructive and non-obstructive causes is essential. The combination of low ejaculate volume, normal hormonal profile, and absence of the vas deferens strongly suggests an obstructive etiology, specifically CBAVD. In this context, evaluation for CFTR mutations is recommended [8,9].

While CF is typically diagnosed in childhood with pulmonary and gastrointestinal involvement, CFTR-related disorders represent a spectrum of disease with variable expression. Some individuals may present later in life with isolated manifestations, such as infertility or pancreatitis, without classic respiratory symptoms. In this patient, recurrent pancreatitis preceded the diagnosis of infertility, providing an important clue to systemic involvement [10,11].

The identification of a pathogenic CFTR variant (p.Phe508del) in combination with a TG12T5 allele is particularly relevant. The TG-T tract modulates splicing efficiency of exon 10, and longer TG repeats (such as TG12) in combination with the T5 allele are associated with reduced CFTR function. When present alongside a pathogenic variant, this combination can result in a spectrum of phenotypes ranging from isolated CBAVD to mild CF [12].

From a clinical standpoint, this case underscores several important considerations for urologists. First, infertility may be the initial presentation of a systemic genetic disorder. Second, a thorough physical examination, including assessment of the vas deferens, is critical and may direct further diagnostic evaluation. Third, genetic testing plays a central role not only in confirming the diagnosis but also in guiding counseling regarding reproductive options and potential health implications.

Management of these patients requires a multidisciplinary approach. Assisted reproductive techniques, such as testicular sperm extraction (TESE) combined with intracytoplasmic sperm injection (ICSI), offer the possibility of biological paternity. However, genetic counseling is essential, as there is a risk of transmitting CFTR mutations to offspring [13].

## Conclusions

Obstructive azoospermia may be the first manifestation of a CFTR-related disorder in adult patients. The coexistence of infertility and recurrent pancreatitis should raise suspicion for underlying CFTR dysfunction, even in the absence of classic CF symptoms. Early recognition allows for appropriate genetic evaluation, multidisciplinary management, and informed reproductive counseling.
